# Prognostic Value of Histological Response to Chemotherapy in Osteosarcoma Patients Receiving Tumor-Bearing Frozen Autograft

**DOI:** 10.1371/journal.pone.0071362

**Published:** 2013-08-15

**Authors:** Shinji Miwa, Akihiko Takeuchi, Hiroko Ikeda, Toshiharu Shirai, Norio Yamamoto, Hideji Nishida, Katsuhiro Hayashi, Yoshikazu Tanzawa, Hiroaki Kimura, Kentaro Igarashi, Hiroyuki Tsuchiya

**Affiliations:** 1 Department of Orthopaedic Surgery, Kanazawa University School of Medicine, Kanazawa, Japan; 2 Section of Diagnostic Pathology, Kanazawa University Hospital, Kanazawa, Japan; University Hospital of Navarra, Spain

## Abstract

**Background:**

A variety of surgical procedures are now available for tissue reconstruction after osteosarcoma excision, and an important prognostic factor is the evaluation of response to chemotherapy using histology. Although tumor-bearing autografts are useful tools for reconstruction, re-use of the primary tumor may make it difficult to assess the histological response to chemotherapy, since the entire tumor cannot be analyzed. Here, we analyzed the prognostic value of the histological response in the patients who received frozen tumor-bearing autografts for reconstruction.

**Method:**

Retrospective analysis of the medical records of 51 patients with high-grade osteosarcoma of the extremities was performed. All patients received reconstruction using frozen tumor-bearing autografts. Tumor necrosis was evaluated in extraskeletal masses and cancellous bone.

**Results:**

Five-year overall survival of patients with good and poor response to chemotherapy was 82.9% and 46.4%, respectively (*P* = 0.044), and 5-year event-free survival was 57.7% and 36.0%, respectively (*P* = 0.329). Multivariate analysis revealed that a poor histological response to chemotherapy was a significant prognostic factor for overall survival (*P* = 0.033).

**Conclusion:**

Histological response is an important and reliable prognostic factor in patients undergoing reconstruction using frozen tumor-bearing autografts.

## Introduction

Before the introduction of chemotherapy, patients with osteosarcoma had an extremely poor prognosis and limb amputation was the standard treatment. One of the purposes of chemotherapy is the eradication of metastatic lesions; however, it is difficult to eradicate all tumor cells when metastatic lesions are clinically detectable. Therefore, metastatic disease at initial diagnosis is one of the most important prognostic factors.

The introduction of chemotherapy markedly stimulated the development of surgical procedures for osteosarcoma. Although mega-prosthesis is currently the most widely used reconstruction procedure after tumor excision, allograft, autograft, allograft-prosthetic composites, autograft-prosthetic composites, distraction osteogenesis, and rotationplasty have also been performed for bone reconstruction [1]–[8]. There are various kinds of autografts including iliac bone, vascularized fibula, and tumor-bearing bone grafts. For use in reconstruction, tumor-bearing bone requires pre-treatment to eliminate viable tumor cells, such as autoclaving, pasteurization, irradiation, or freezing in liquid nitrogen. In our institute, frozen bone autografts have been used for reconstruction following malignant bone tumor excision since 1999 [6]. Reconstruction using tumor-bearing bone creates a problem with assessment of chemotherapeutic effects, which is important information required for evaluating the chemotherapy treatment plan including the number of courses and anticancer drug choice, surgical procedure, and surgical margins. Chemotherapeutic effects are commonly assessed by comparing the cellularity and tumor necrosis of a biopsy specimen and resected bone. While a large area of the tumor can be assessed in cases of amputation and mega-prosthesis, only a part of the tumor is available in cases of reconstruction using tumor-bearing bone. The latter is assumed to decrease the accuracy of assessments of chemotherapeutic effects; however, this has not been analyzed objectively. Here, we have analyzed the accuracy and prognostic value of tumor necrosis following reconstruction using frozen tumor-bearing autografts.

## Methods

### Patients

A total of 59 patients with osteosarcoma were treated with reconstruction using frozen tumor-bearing autografts between January 1999 and November 2011; patients with low-grade osteosarcoma, truncal site, or non-surgical patients were excluded. This study was approved by the Institutional Review Board of the Kanazawa University Graduate School of Medical Science, Kanazawa, Japan. Written informed consent was obtained from all patients and/or their family.

All patients received chemotherapy according to the K2 protocol [6], which was modified for each patient based on their general condition, past history of chemotherapy, renal and liver functions. Three courses of chemotherapy using cisplatin (120 mg/m^2^), doxorubicin (30 mg/m^2^×3 days) and caffeine (1.5 g/m^2^×3 days) were administered at 3-week intervals. The effects of chemotherapy were evaluated radiologically after three courses of chemotherapy. An additional two courses of chemotherapy were administered to good responders. Poor responders either underwent surgery immediately or were given other drugs such as ifosfamide or etoposide. After surgical treatment, patients received 3–6 additional courses of intravenous cisplatin, doxorubicin, and caffeine. The primary tumor, local recurrence, and metastasis were assessed clinically and radiologically examined using X-ray, CT, MRI, and bone scintigraphy.

### Surgical Procedure

**Figure 1:**
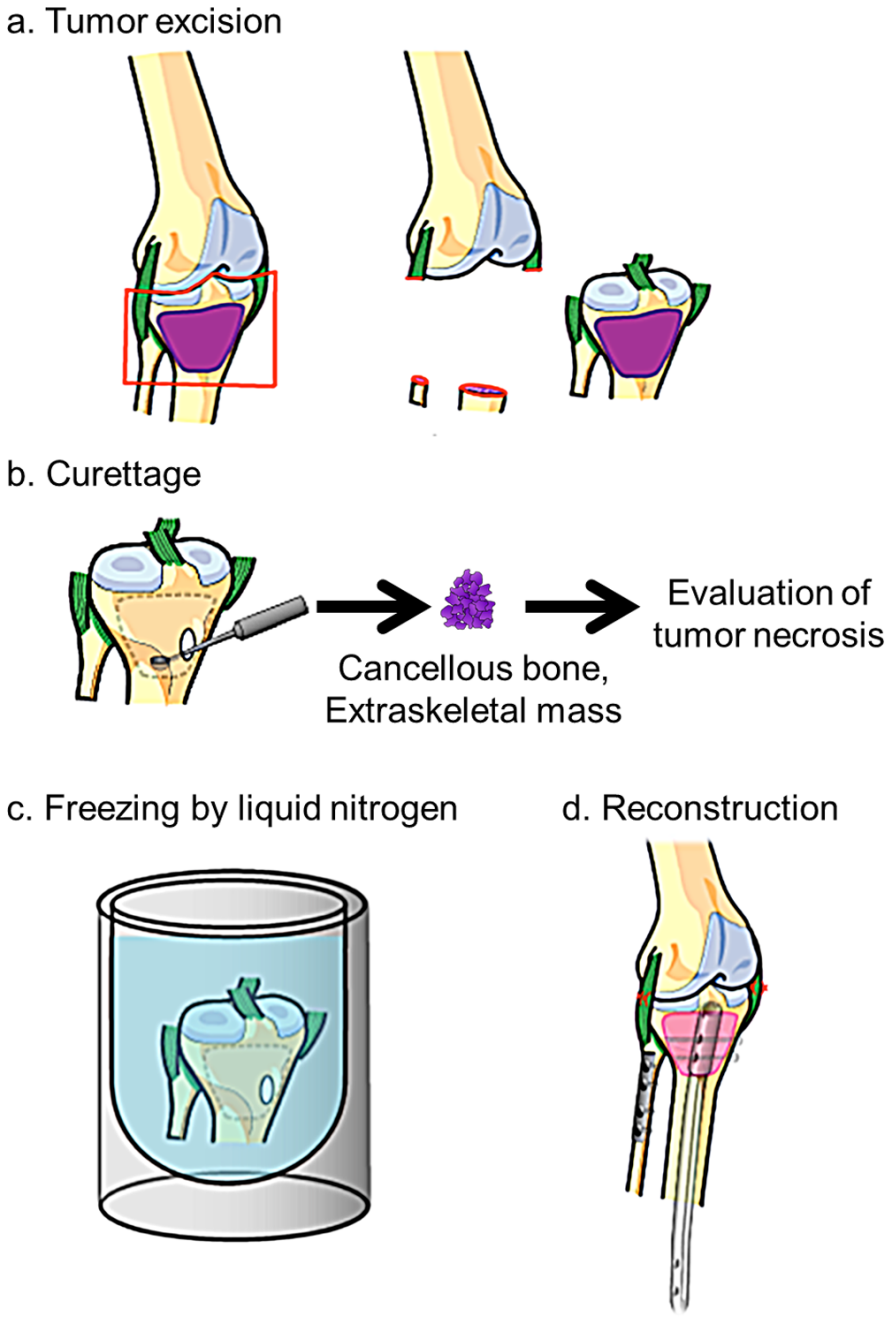
Procedure of reconstruction with frozen autograft and assessment of chemotherapeutic effect.

Frozen tumor-bearing autografts were obtained by tumor excision, curettage, freezing in liquid nitrogen, and used in reconstruction (Fig. 1) [7].

#### a. Tumor excision

The tumor was excised with a safety margin.

#### b. Curettage

Soft tissues were removed from the excised tumor-bearing bone. After cancellous bone was curetted, excess water in the bone was removed by suction to prevent bone damage due to ice expansion during freezing. Curetted cancellous bone and extraskeletal masses were evaluated histologically.

#### c. Freezing by liquid nitrogen

The tumor-bearing bone was frozen in liquid nitrogen for 20 min, thawed in air at room temperature for 15 min, followed by thawing in distilled water for 10 min.

### Reconstruction

The massive bone and osteochondral defects following resection were reconstructed using frozen tumor-bearing autografts and instruments such as prostheses, intramedullary nails, and plates.

### Histological Evaluation of Chemotherapeutic Effect

Histological responses to chemotherapy were assessed by comparing tumor biopsy and resected specimens, including cancellous bone, as well as extraskeletal masses not requiring reconstruction (Fig. 1). All the curetted tumors and the extraskeletal masses including largest cross-section were observed to assess the response to chemotherapy. Histologic gradings were based on the degree of necrosis: grade IV (100% necrosis), grade III (90–99% necrosis), grade II (50–89% necrosis), and grade I (0–49% necrosis) [9]. Grade III and IV were classified as good responses to chemotherapy; grade I and II were poor responses.

### Statistical Analysis

We examined the prognostic significance of age (<40 years vs. ≥40 years), gender, histological subtype (osteoblastic type or others), metastatic disease at initial diagnosis, and histological response (grade I and II vs grade III and IV). Overall survival and event-free survival were calculated using the Kaplan–Meier method with log-rank test. Multivariate Cox proportional hazards regression analyses were used to identify independent predictors of overall survival and event-free survival, which were defined as the time from the initial diagnosis to death from any cause and time from the initial diagnosis to metastasis, local recurrence, or death from any cause. Statistical significance was defined as *P*<0.05; factors with *P*<0.4 in univariate analysis were included in multivariate Cox proportional hazards models. Statistical analyses were performed with EZR (Saitama Medical Center, Jichi Medical University).

## Results

### Patients

**Figure 2:**
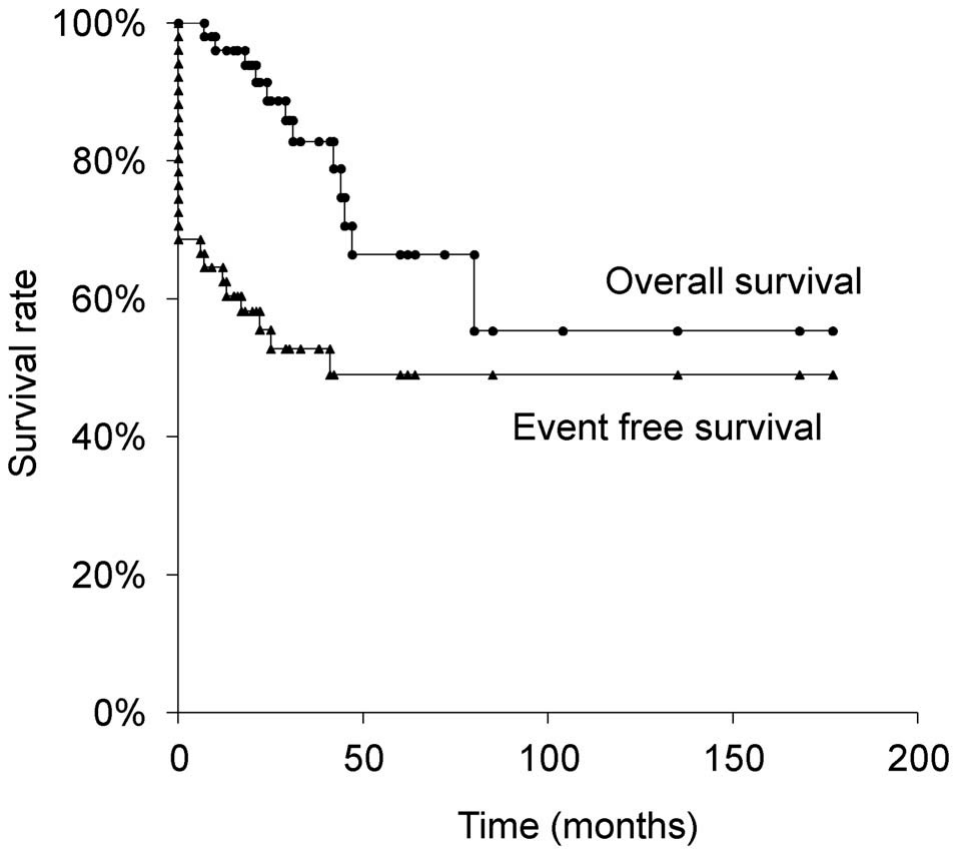
Overall and event-free survival of all 51 patients.

A total of 51 osteosarcoma patients receiving frozen tumor-bearing autografts were retrospectively reviewed, consisting 28 male and 23 female patients with a mean age of 21.2 years (range, 5–69) (Table 1). The mean follow-up period was 44.6 months (range, 7–177). At initial diagnosis, 16 patients (31.4%) had clinically detectable metastases, while the rest did not. Histologically, 37 were osteoblastic type, 10 were chondroblastic type, 2 were fibrous type, and 2 were the other type. The 3-, 5-, and 10-year overall survival rate of the study patients was 82.8%, 66.4%, and 55.3%, respectively (Fig. 2). The 3-, 5-, and 10-year event-free survival rate was 52.8%, 49.0%, and 49.0%, respectively (Fig. 2). In histological evaluation of chemotherapeutic effects, 21 patients were grade IV, 15 patients were grade III, 13 patients were grade II, and 2 patients were grade I. On the basis of the Enneking’s surgical staging, 2 patietns were classified as stage IIA, 33 as stage IIB, and 16 as stage IIIB [10].

**Table 1:** Characteristics of the study patients.

Characteristic	
Follow up period (months)	44.6 (7–177)
Age at diagnosis (y)	21.2 (5–69)
<40	44
≥40	7
Gender	
Male	28
Female	23
Primary tumor location	
Femur	30
Tibia	16
Humerus	3
Fibula	1
Calcaneus	1
Pathologic subtype	
Osteoblastic	37
Chondroblastic	10
Fibroblastic	2
Other	2
Surgical stage	
IIA	2
IIB	33
IIIA	0
IIIB	16
Huvos grade	
I (0–49% necrosis)	2
II (50–89% necrosis)	13
III (90–99% necrosis)	15
IIIB (100% necrosis)	21

### Overall Survival

**Figure 3:**
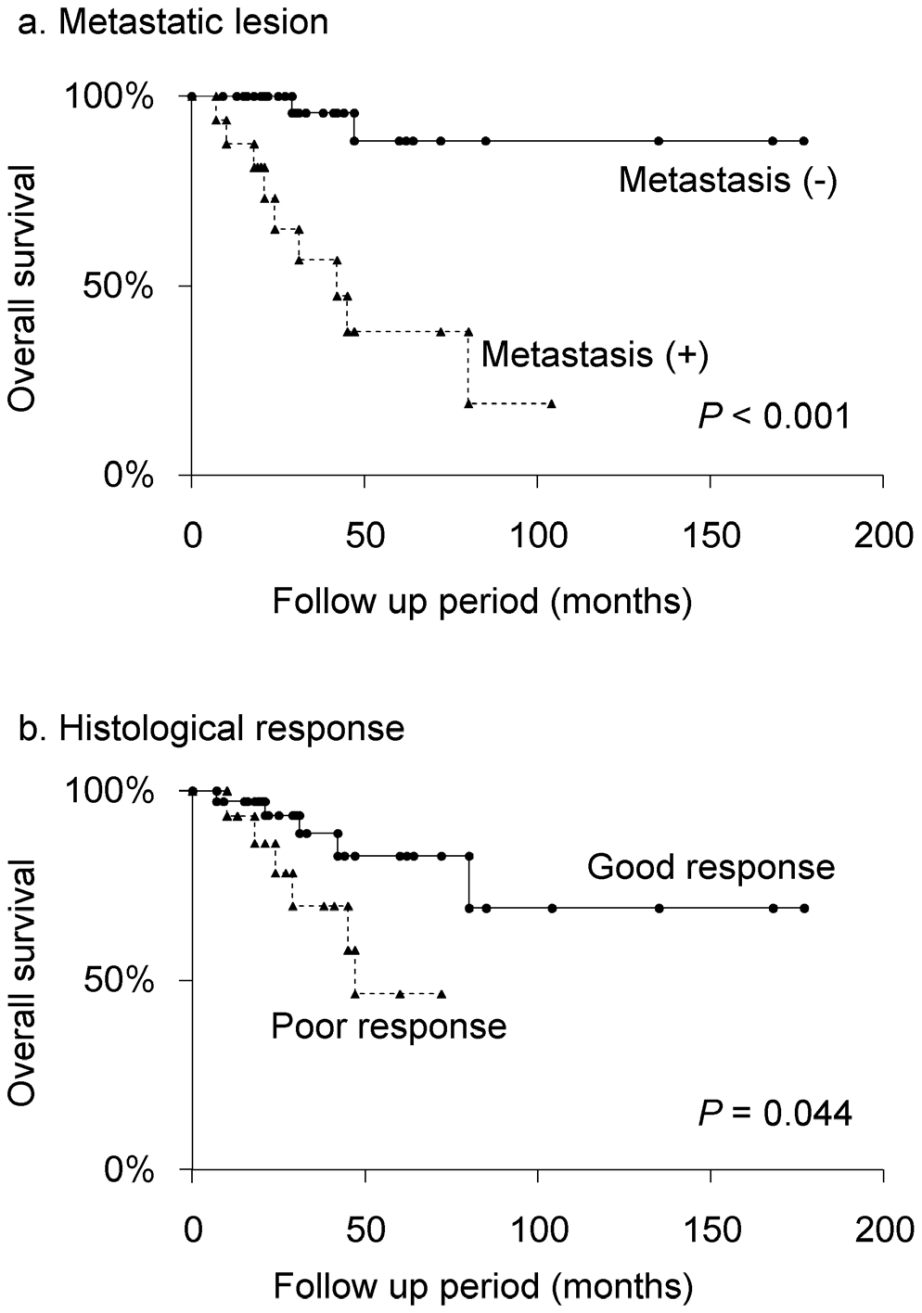
Kaplan-Meier curves of overall survival for variables with prognostic significance in univariate analyses. a. Metastatic lesion. b. Histological response.

Kaplan–Meier analysis revealed that absence of metastasis at initial diagnosis was significantly associated with good overall survival in the patients (*P*<0.001; Table 2). The 5-year overall survival of patients with or without metastases was 37.9% and 88.3%, respectively (Fig. 3a). Histological response was also significantly associated with good overall survival (*P* = 0.044; Table 2). The 5-year overall survival rate of poor responders (less than 90% necrosis) was 46.4% compared with 82.9% in good responders (90% or more necrosis) (Fig. 3b). There were no significant associations between overall survival and age, gender, or pathological tumor type.

**Table 2:** Univariate analysis of overall survival.

Factor	Patient group	5-year survival	*P*
Age (≥40)	<40	68.8%	0.845
	≥40	75.0%	
Male	Male	85.9%	0.079
	Female	46.0%	
Pathological type	Osteoblastic	58.9%	0.078
	Other type	100.0%	
Metastatic disease	Metastasis (−)	88.3%	<0.001**
	Metastasis (+)	37.9%	
Histological response	Good	82.9%	0.044*
	Poor	46.4%	

* *P*<0.05,

** *P*<0.01.

Multivariate analysis showed that metastatic disease at initial diagnosis was the most important independent predictor of overall survival (HR, 8.33; CI, 1.9–36.1; *P* = 0.005), followed by poor histological response (HR, 3.98; CI, 1.1–14.1; *P* = 0.033; Table 3).

**Table 3:** Multivariate Cox models of overall survival for extremity osteosarcoma treated with tumor-bearing frozen autograft.

Charasteristic	HR	95%CI	*P*
Male	0.74	0.20–2.81	0.661
Osteoblastic type	0.57	0.06–5.18	0.617
Histological poor response	3.98	1.12–14.12	0.033*
Metastatic disease	8.33	1.93–36.06	0.005**

* *P*<0.05,

** *P*<0.01.

### Event Free Survival

The presence of metastases at initial diagnosis (*P*<0.001) correlated to a significant degree with event-free survival (Table 4): the 5-year event-free survival of metastatic patients was 0%, compared with 69.4% for non-metastatic patients. There were no associations between event-free survival and age, gender, pathological type, and histological response to chemotherapy.

**Table 4:** Univariate analysis of event free survival.

Factor	Patient group	5-year survival	*P*
Age (≥40)	<40	50.1%	0.581
	≥40	53.6%	
Male	Male	56.1%	0.258
	Female	41.9%	
Pathological type	Osteoblastic	42.3%	0.329
	Other type	64.3%	
Metastatic disease	Metastasis (−)	69.4%	<0.001**
	Metastasis (+)	0%	
Histological response	Good	57.7%	0.329
	Poor	36.0%	

** *P*<0.01.

Multivariate analysis showed that metastatic disease at initial diagnosis was the only important independent predictor of event-free survival (HR, 15.66; CI, 4.8–51.5; *P*<0.001; Table 5). Histologically, poor response (necrosis <90%) did not show significant correlation with event-free survival (HR, 1.72; CI, 0.7–4.1; *P* = 0.217).

**Table 5:** Multivariate Cox models of event free survival for extremity osteosarcoma treated with tumor-bearing frozen autograft.

Charasteristic	HR	95%CI	*P*
Male	0.58	0.24–1.41	0.227
Osteoblastic type	1.76	0.55–5.61	0.341
Histological poor response	1.72	0.73–4.09	0.217
Metastatic disease	15.66	4.75–51.53	<0.001**

** *P*<0.01.

### Prognostic Value of Histological Response to Chemotherapy in Patients with or without Metastasis

**Figure 4:**
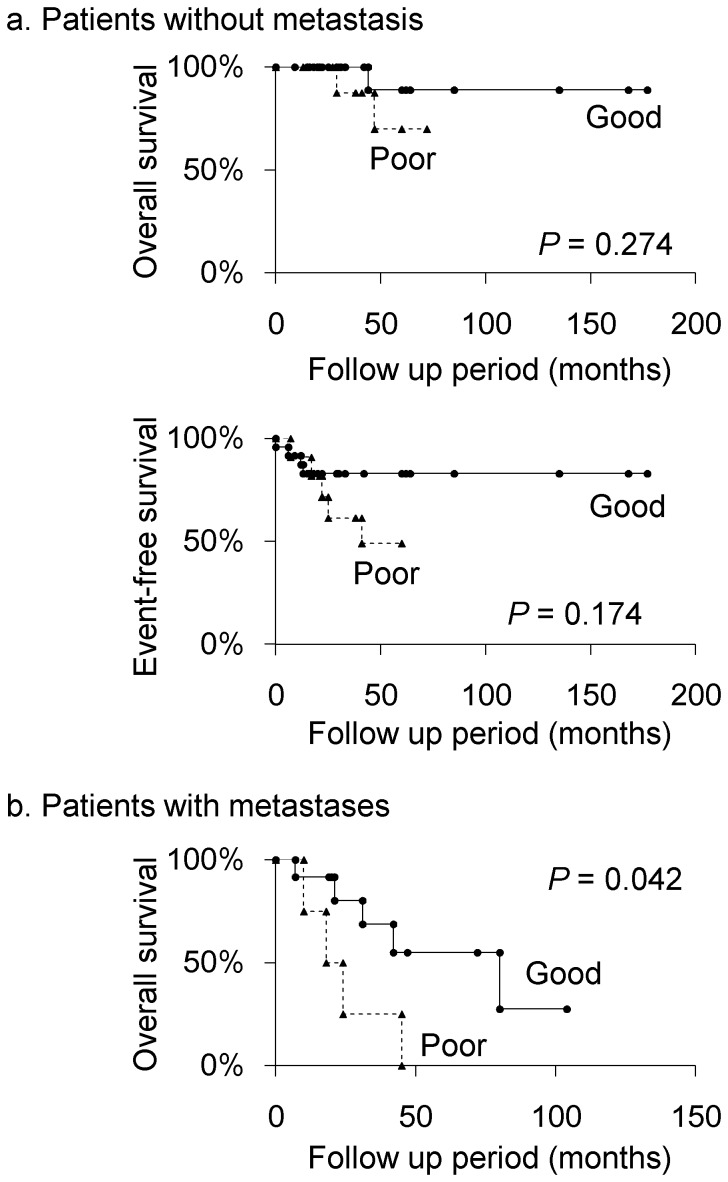
Kaplan-Meier curves of overall survival and event-free survival for histological response in patients with or without metastasis. a. Patients without metastasis. b. Patients with metastases.

The study patients were divided into the patients without metastasis and the patients with metastases, and the prognostic values of histological response to chemotherapy were evaluated in each group. Among the patients without metastasis, 5-year overall survival rates of good responders and poor responders were 88.9% and 70.0%, respectively (*P* = 0.274; Fig. 4a). Five-year event-free survival rates of good responders and poor responders were 82.9% and 49.1%, respectively (*P* = 0.174; Fig. 4a). Among the patients with metastases, 3-, 5-year overall survival rates of good responders were 68.8% and 55.0%, compared with 25.0% and 0% in poor responders (*P* = 0.042; Fig. 4b).

## Discussion

The proportion of limb salvage surgery in the treatment of osteosarcoma has increased since the 1980s. Currently, there are a variety of options of reconstructive procedures, including osteoarticular bone grafts, intercalary bone graft, autograft- or allograft-prosthetic composites, and mega-prosthesis [1]–[8], [11]–[14]. Although mega-prosthesis is a useful and commonly used reconstructive tool, tumor-bearing autograft is also useful for the reconstruction of massive bone and osteochondral defects following the resection of osteosarcoma. Although allografts are difficult to obtain in Asian countries for religious reasons, tumor-bearing autografts are readily available and have no compatibility problems. Before being used for reconstructive surgery, bone grafts must undergo one of several pre-treatments, including pasteurization, autoclaving, irradiation, or freezing in liquid nitrogen; the latter is particularly advantageous owing to its simplicity and rapidity [7], [8]. Furthermore, freezing maintains tissue microstructure and tumor antigens, whereas pasteurization and irradiation cause protein degradation [15]. Consequently, frozen autografts are associated with good bone induction and conduction as well as activation of tumor immunity [16]–[18]. Although many techniques for re-using tumor-bearing bone need osteotomy, which may cause non-union, this disadvantage is not associated with pedicle freezing since this can be performed without osteotomy [7], [8].

Histological response to chemotherapy is one of the most important prognostic factors for patients with osteosarcoma [19], [20]. The histological response to chemotherapy usually influences post-operative treatment, including possible changes of anticancer drugs and indicating the need for additional treatments such as radiation therapy. Many experimental studies have been reported the use of drug-response assay. Histoculture drug response assay (HDRA), which cultures three-dimensional tumor tissue, highly correlates with histological response to chemotherapy [21], [22]. Furthermore, there are reports which describing that chemosensitivity determined by the HDRA seems to be a strong predictor of survival in patients with malignant tumors [23]–[25]. Although there are few reports describing the use of HDRA for osteosarcoma, HDRA would be complementary to evaluation of chemotherapeutic effects. On the other hand, we previously reported that the chemotherapeutic effects evaluated by ^99m^Tc-MIBI scintigraphy (^99m^Tc-MIBI) and combined radiological score (CRS) had significant correlation with histological response to chemotherapy [26], [27]. Furthermore, the chemotherapeutic effects evaluated by^ 99m^Tc-MIBI and CRS correlated with overall survival [28]. Although chemotherapeutic effects are important information to make the surgical treatment plan, it is impossible to assess histological response before tumor excision. These reports suggest that ^99m^Tc-MIBI and CRS could be used as supplementary tool for assessment of chemotherapeutic effects before surgical treatment.

Histological responses to chemotherapy are commonly evaluated by comparing necrosis of the resected tumor with the biopsy specimen. In our institute, chemotherapeutic effects have been evaluated according to Huvos grading system, one of the most standard grading systems [9]. Many studies have shown that the histological response to preoperative chemotherapy is one of the most important predictors for clinical outcome of osteosarcoma [19], [29]–[34]. In most of these studies, less than 90% of chemotherapy induced tumor necrosis rate was graded as “poor”, and 90% or more tumor necrosis rate was defined as “good”. Compared with poor responders, good responders had significantly higher event-free survival [27], [29], [32] and/or overall survival [19], [29]–[34]. However, the use of 90% necrosis as a cutoff point in assessment of chemotherapeutic effect is controversial [35], [36]. Some studies showed that histological response was not associated with survival of osteosarcoma patients [37]–[39]. Li et al. reported that tumor necrosis grouped at 90% was not associated with OS and EFS, while patients with greater than 70% necrosis rate had significantly higher EFS than those with less than 70% [35]. Harting reported that patients with less than 50% necrosis rate had significantly poor OS [36]. In further study, several cutoff points of tumor necrosis should be analyzed to define the adequate cutoff point.

Tumor cellularity and the degree of necrosis due to chemotherapy are heterogeneous throughout the tumor. An unsubstantiated disadvantage of using tumor-bearing autografts is that the histological assessment of the small, pre-reconstruction sample from the graft may not be representative of the cellularity and necrosis of the tumor as a whole. Here, we have addressed this important unknown by analyzing the correlation between histological response and prognosis in osteosarcoma patients who underwent reconstructive surgery using frozen tumor-bearing autografts. We found that the histological response determined from the graft biopsy was an important prognostic factor in these patients, indicating that this analysis was adequate and representative of the whole tumor. Our study only included patients who received frozen autografts; therefore, the accuracy of assessing the chemotherapeutic effect in the patients who receive tumor-bearing autograft including irradiated bone, pasteurized bone, and autoclaved bone should be discussed. Our data indicate that histological response to chemotherapy is also a reliable and important prognostic factor in osteosarcoma patients who received tumor-bearing autografts for reconstruction.

There are several limitations in our study. First, the patient numbers of the present study was small because osteosarcoma has low incidence in the whole population (2–3/million/year) [40]. Secondly, we did not compare the prognosis and histological responses to chemotherapy of patients receiving tumor-bearing autografts vs. a control group. Although there were patients who received mega-prosthesis or limb amputation during the study period, and their histological responses to chemotherapy could have been assessed by total tumor specimens, these patients were not sufficiently similar to be used as controls, e.g., based on tumor size, invasion into nerve or vessels, all of which may influence their overall and event-free survival. Therefore, it is unclear whether assessing histological response to chemotherapy using a small sample is an accurate analysis of the entire tumor, which would require a prospective and/or direct comparison of the different patient groups. Further study with more patients should also be performed to evaluate the predictive value of the tumor necrosis in patients receiving tumor-bearing autograft.

### Conclusions

Histological response to chemotherapy and metastasis at diagnosis were both significantly correlated with overall survival. The correlation between the histological response to chemotherapy and overall survival suggested its accuracy as a prognostic factor for patients receiving tumor-bearing autografts for tissue reconstruction.
